# Evolutionary Impacts of Pattern Recognition Receptor Genes on Carnivora Complex Habitat Stress Adaptation

**DOI:** 10.3390/ani12233331

**Published:** 2022-11-28

**Authors:** Xiaoyang Wu, Jun Chen, Xibao Wang, Yongquan Shang, Qinguo Wei, Honghai Zhang

**Affiliations:** 1 College of Life Sciences, Qufu Normal University, Qufu 273165, China; 2 College of Marine Life Sciences, Ocean University of China, Qingdao 266005, China

**Keywords:** Carnivora, evolution, immune response, niche, pattern recognition receptors

## Abstract

**Simple Summary:**

Carnivora is a complex and diverse group, and its members are considered the most proficient carnivores in nature. However, the evolutionary impacts of pattern recognition receptor (PRR) genes on Carnivora’s stress adaptation to complex habitats are poorly understood. In this study, we explored the evolution of 946 PRR gene sequences in 43 Carnivora species to elucidate the molecular mechanisms of carnivore adaptation to complex habitats. We found that the PRRs were relatively conserved, and different gene families showed different evolutionary patterns. PRRs were highly purified based on their overall roles in Carnivora species but interspersed with positive-selection patterns during evolution. In addition, the selection pressure of toll-like receptor 10 was relaxed in seven species with pseudogenes, which may have emerged during recent evolutionary events. Our findings offer valuable insights into the molecular and functional evolution of PRR genes, which are important for immune adaptations in Carnivora.

**Abstract:**

Many mammals develop specific immune responses owing to the changes in their ecological niche and diet that are essential for animal survival. However, pattern recognition receptors (PRRs) serve as the first line of defense in innate immunity and generate immune responses in the host. However, the evolutionary impacts on PRR genes in Carnivora are not well studied. Herein, we explored the evolution of 946 PRR gene sequences in 43 Carnivora species to elucidate the molecular mechanisms of carnivore adaptation to complex habitats. We found that the PRRs were relatively conserved, and different gene families showed different evolutionary patterns. PRRs were highly purified based on their overall roles in Carnivora species but interspersed with positive-selection patterns during evolution. Different niche types may have jointly driven the evolution of PRR genes. In particular, the selection pressure of toll-like receptor (TLR) 10 was relaxed in seven species with pseudogenes, which may have emerged during recent evolutionary events. We speculated that a “functional compensation” mechanism may exist for genes with overlapping functions in the TLR gene family. Additionally, *TLR2*, *TLR4*, NLRC5, and DECTIN1 were subject to positive selection in semi-aquatic species, and the adaptive evolution of these genes may have been related to the adaptation to semi-aquatic environments. In summary, our findings offer valuable insights into the molecular and functional evolution of PRR genes, which are important for immune adaptations in Carnivora.

## 1. Introduction

Previous research has identified over 250 species of surviving Carnivora [1], a taxonomic class with remarkable biological diversity. Carnivora evolved from Miacis approximately 42 million years ago and rapidly diverged into Feliformia and Caniformia [2]. During evolution, the members of both suborders have adapted to different environments (including land, fresh water, and oceans) and differentiated to adapt to different lifestyles, such as living in groups, living alone, and living in caves. In addition, during a long period of adaptive evolution, Carnivora species adapted to their own carnivorous needs in terms of morphological structure and physiology, occupying complex ecological niches with different lifestyles (semi-aquatic and terrestrial). Moreover, their evolution in various types of ecological niches makes carnivores a good model for studying the molecular mechanisms of disease resistance and environmental adaptation.

Pattern recognition receptors (PRRs), as the first line of defense of innate immunity, generate host immune responses by recognizing pathogen-associated molecular patterns (PAMPs). The function of PRRs is to monitor the presence of viral molecules, initiate the signaling pathways of inflammation and antiviral immunity in the body, and protect the host from infections [3]. Viruses invade host cells and generate new viral genomes using base components in the cells, forming major PAMPs of viral nucleic acid, which can be identified by PRRs [4]. PRRs include the toll-like receptors (TLRs), RIG-I-like receptors (RLRs), NOD-like receptors (NLRs), and C-type lectin receptors (CLRs) [5]. PRRs induce the production of cytokines and interferons by activating related anti-inflammatory pathways, thereby triggering the body’s antiviral response [4]. In previous studies, monitoring viral DNA in infected cells by PRRs has been extensively studied [6]. Studies have revealed that RNA polymerase III transcribes some viral DNA to RNA, which is identified by the host’s *RIG-I* recognition receptors and induces type I interferon (IFN) production [4]. PRRs identify highly conserved molecular structures that are common on the surface of pathogenic microorganisms. For example, *TLR3* identifies dsRNA [7], and *TLR4* identifies lipopolysaccharides (LPSs) [8]. The recognition pattern of PRRs is not strictly one-to-one correspondence; for example, *TLR4* can also identify certain viral proteins [9]. However, few reports have described the three families of innate immune-recognition receptor genes in the Carnivora species.

Recently, with the rapid development of high-throughput sequencing technologies, an increasing amount of genomic information of the Carnivora species has been analyzed, thereby providing a strong basis for studying their adaptive evolution. During the transition from terrestrial to aquatic environments, carnivores gradually adapt to the threat of pathogenic microorganisms in different habitats, although the underlying adaptive immune mechanisms remain unclear. Therefore, in this study, we systematically investigated the adaptive evolution of PRR genes in 43 Carnivora species. These data will improve our understanding of the innate immune system of carnivorous species at the molecular level and provide an immunological basis for a comprehensive understanding of carnivore adaptation to semi-aquatic environments.

## 2. Materials and Methods

### 2.1. Taxon Coverage and Dataset Preparation

Our dataset encompassed genome assemblies of 43 mammal Carnivora species. All available genome sequences for Carnivora species were collected from the National Center for Biotechnology Information (NCBI; https://www.ncbi.nlm.nih.gov/, accessed on 13 December 2019), Ensemble (http://www.ensembl.org/index.html, accessed on 13 December 2019), and GigaDB (http://gigadb.org/, accessed on 13 December 2019) databases, and the genomes were classified as annotated and unannotated genomes. All genomic information is presented in Table S1.

### 2.2. Identification of Pattern Recognition Receptor Genes

**Figure 1:**
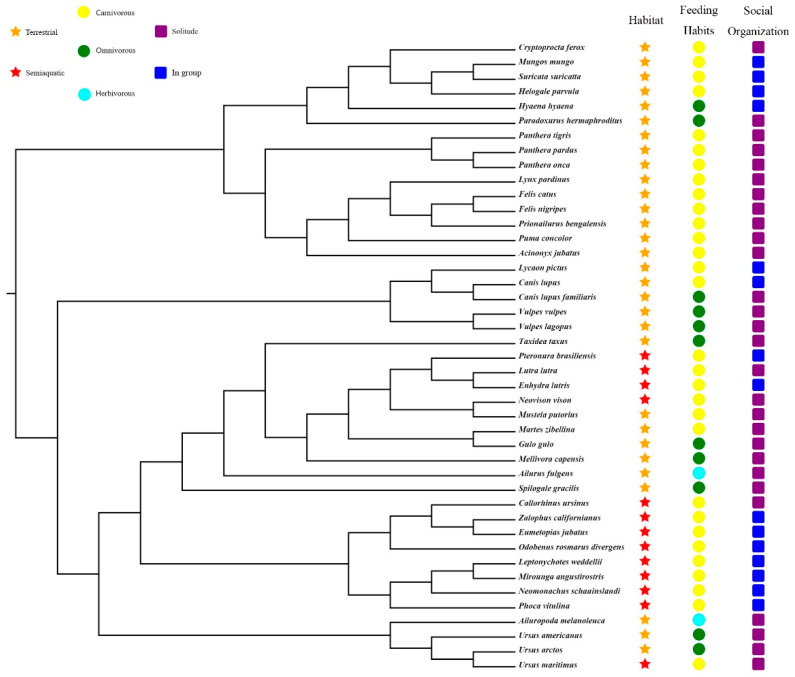
Phylogenetical relationships, niche classification, feeding habits, and social organization of 43 carnivore species in this study (https://www.iucnredlist.org/, accessed on 1 June 2019) [13,14,15].

First, we downloaded the nucleotide sequences of PRRs of all annotated Carnivora species from the NCBI database. Subsequently, the unannotated Carnivora genomes were screened using local BLAST software to construct a local genome database [10]. The protein-coding sequences of dog and domestic cat PRRs were then used as seed sequences (Table S2), and tblastn (E-value cutoff of 10^−5^) was used to search for PRR sequences in the unannotated genomes, following which the regions in the locked genes were extracted using TBtools software [11], and the target sequences were obtained from the GeneWise (https://www.ebi.ac.uk/Tools/psa/genewise/, accessed on 1 June 2022) website. Subsequently, MEGA X software [12] was used to align the obtained target sequences with the seed sequence. If a target sequence had deletions, then the regions of the seed sequence corresponding to the missing regions were appropriately extended and extracted as a new sequence, which was used to perform a BLASTN search for the target genome to fill in the missing sequence of the target gene. Finally, based on the characteristics of the obtained target genes, the obtained PRR gene sequences of all species were further divided into intact genes, partial genes, pseudogenes, and absent genes. The phylogenetical relationships, niche classification, feeding habits, and social organization of 43 carnivore species in this study are shown in Figure 1.

### 2.3. Phylogenetic Tree Construction

We used MEGA X software to convert the obtained target nucleotide sequences into amino acid sequences and then used Multiple Sequence Comparison by Log-Expectation software to compare the obtained amino acid sequences [16]. Subsequently, the aligned sequences were exported, and Gblocks software was used to extract the conserved sites in the alignment results of multiple sequences [17]. A maximum likelihood (ML) tree was built using IQ-TREE software [18]. Using the IQ-TREE checking tool and a bootstrap value of 1000 replicates, we obtained a tree with a high likelihood value that was based on the optimal model recommended by the Bayesian information criterion. Finally, iTOL v4 software [19] was used to visualize and quantify the ML tree.

### 2.4. Molecular Evolutionary Analyses

Nonsynonymous substitutions (dN) and synonymous substitutions (dS) of genes were calculated and compared using the “codeml” program of PAML software (v.4.7) [20]. ω (the dN:dS ratio) was used to measure the evolution rates of genes. The guide tree for PAML analysis was obtained from the TimeTree website (http://www.timetree.org/, accessed on 10 May 2021).

The site model allows for the ω values of different sites in the same sequence to change. We used two models to detect the positive selection of different sites in each PRR gene, namely the M7 (null hypothesis: 0 < ω < 1) and M8 (alternative hypothesis: ω > 1) models. In the result of the likelihood-ratio test (LRT) of the two models, if the *p*-value of LRT is not significant, the null hypothesis M7 model is accepted; otherwise, the alternative hypothesis M8 model (positive choice model) is accepted. In the case of a significant model, the positive-selection sites detected in the M8 model can be calculated by applying the method of Bayes empirical Bayes (BEB). When the posterior probability is greater than 0.90, this site is considered to be a potential positive-selection site.

We also utilized three ML methods with the online Datamonkey Adaptive Evolution Server (https://www.datamonkey.org/, accessed on 10 May 2021) to identify positively selected sites. The three ML methods used were the single likelihood ancestor counting, fixed-effect likelihood, and random-effect likelihood models. In addition, we used all three ML methods in Datamonkey to assist the M8 model in screening for positive-selection sites, stipulating that those sites identified with at least two ML methods screened at the same time were considered positive-selection sites. The screening criteria for positive-selection sites were *p*-value less than 0.1 for SLAC and FEL and Bayes Factor greater than 50 for REL.

The branch model employs a combination of a one-ratio (M0) model and a free-ratio (M1) model to detect adaptive evolution among different lineages, based on the LRT algorithm of the CODEML program. To detect selection pressure on different clades of Carnivora species, the results of both models were compared using the likelihood test, and clades showing evidence of positive selection were marked on the phylogenetic tree.

The branch-site model is primarily used to detect the effects of positive selection in foreground branches at some sites. Using this model, modified model 1 (Ma: Model = 2, NSsites = 2) was compared with the null model (Fix_omega = 1, Omega = 1). When the significant positive selection was detected with the branch-site model, the empirical Bayes method was used to calculate the posterior probabilities of the positive-selection sites. When the posterior-probability value of a site was greater than 0.90, it was considered to have undergone positive selection.

The Clade C (CmC) model (Model = 3, NSsites = 2) is a type of clade model that allows exploring different evolution rates between different groups [21]. To explore the evolutionary rates of PRR genes in different ecological niches, 43 carnivores were classified into three groups based on their niches (Figure 1): habitat (semi-aquatic or terrestrial), food (herbivorous, omnivorous, or carnivorous), and population size (solitary or gregarious). We used the CmC model to test an alternative hypothesis for comparison purposes with the M2a_ref model of the null hypothesis and calculated gene-evolution rates based on two partitions for habitat (semi-aquatic or terrestrial), three partitions for food (herbivorous, carnivorous, and omnivorous), and two partitions for the population size (solitary and social). The initial ω values were set to 0.5, 1, and 1.5, respectively.

### 2.5. Identifying Functional Domains and Predicting Three-Dimensional Protein Structures

Based on the above analysis of positively selected gene sites, we considered the sites that were detected using at least two ML methods as potential positively selected sites. To determine whether the obtained positive-selection sites of PPR genes were located on important functional domains of the protein structure, we used the Eurasian otter gene as the reference sequence. Important domains of all PRR genes were confirmed using SMART [22] (http://smart.embl-heidelberg.de/, accessed on 10 May 2022), and the I-TASSER website [23] (https://zhanglab.ccmb.med.umich.edu/I-TASSER/, accessed on 10 May 2022) was used to predict the three-dimensional protein structure of each gene. Finally, the candidate positive-selection sites were annotated in the three-dimensional protein structure using PyMOL (https://pymol.org, accessed on 10 May 2022).

## 3. Results

### 3.1. Acquisition of PRR Genes by Carnivora Species

**Figure 2:**
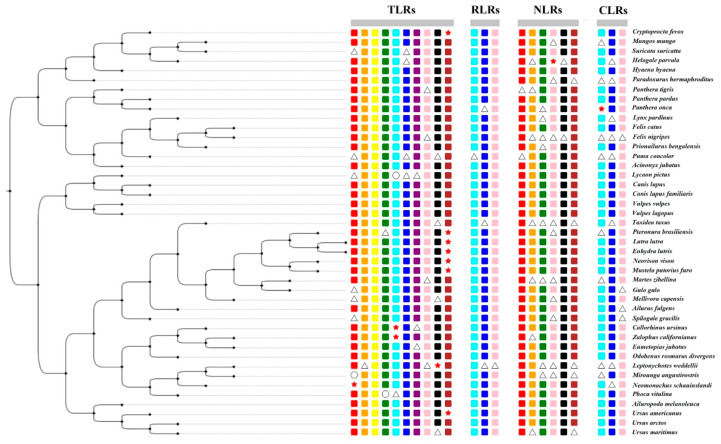
Divergence in PRR genes in the 43 Carnivora species (rectangle, circle, triangle, and star represent intact genes, absent genes, partial genes, and pseudogenes, respectively).

We selected 25 PRR genes from 43 Carnivora species, including 10 members of the TLRs (*TLR1-TLR10*), 3 members of the RIG-I-like receptors (*LGP2*, *MDA5,* and *RIG-I*), 9 members of the NOD-like receptors (*NOD1*, *NOD2*, *NLRC3*, *NLRC5*, *NLRX1*, *NLRC4*, *NLRP1*, *NLRP2*, and *CIITA*), and 3 members of the C-type lectin receptors (*MRC1*, *MRC2,* and *DECTIN1*). Due to the low number of three genes (*NLRC4*, *NLRP1*, *NLRP2)*, we discarded them in the subsequent analysis. The result is that we obtained 22 PRR genes (out of 946 gene sequences) from 43 Carnivora species. Sequences with frameshift mutations and premature stop codons were considered to represent potential pseudogenes. If a gene was not found through a keyword search and a local BLAST, the gene was considered to be missing. If a part of the target sequence was missing after the sequence was compared with the seed sequence, it was considered as a partial gene, and the remaining sequence was determined to find the complete gene (functional gene). The specific acquisition of all sequences is shown in Figure 2 and Figure S1 and Table S3. In this study, we found that *TLR10* might have become a pseudogene in giant otters, sea otters, American minks, ferrets, American black bears, and mongooses.

### 3.2. Phylogenetic Analysis

We constructed phylogenetic trees of the *TLR*, *RLR*, *NLR*, and *CLR* gene families. The best-fit partitioning schemes and corresponding nucleotide substitution models were GTR + T + F5. To enable a more accurate comparison of the topology of the species tree, we discarded pseudogenes and shorter partial genes during the phylogenetic analysis.

**Figure 3:**
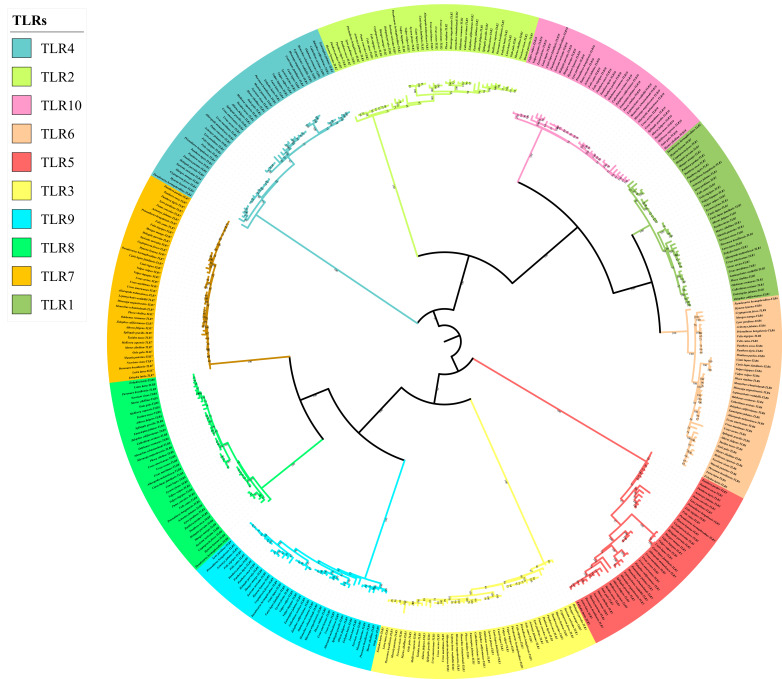
Phylogenetic tree of TLR genes in the 43 Carnivora species.

The phylogenetic tree of the TLR gene family was divided into 10 main branches, where each gene occupied a branch (Figure 3). *TLR1/2/6/10* (*TLR2* subclass), *TLR7/8/9* (*TLR9* subclass), *TLR3* (*TLR3* subclass), *TLR4* (*TLR4* subclass), and *TLR5* (*TLR5* subclass) clustered together or separately. The results were consistent with those of a previous analysis of vertebrate TLR genes, based on sequence similarities [24]. The phylogenetic tree could be divided into two branches: non-viral TLRs (*TLR1/2/4/5/6/10*) and viral *TLRs* (*TLR3/7/8/9*). In addition, we found that the tree structure of each gene was highly consistent with the topology of the species tree; for example, Caniformia (Canidae, Mustelidae, Ailuridae, Ursidae, Odobenidae, Otariidae, and Phocidae) and Feliformia (Felidae, Herpestidae, Hyaenidae, and Viverridae) grouped together.

**Figure 4:**
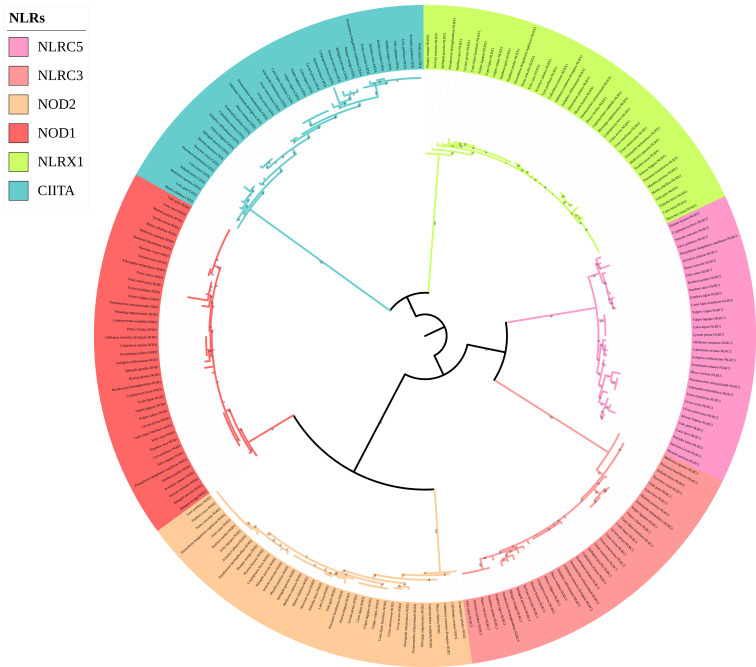
Phylogenetic tree of NLRs genes in the 43 Carnivora species.

The phylogenetic tree of the *NLR* gene family (Figure 4) was divided into six main branches, and each gene was located in a different branch. Four genes (*NOD1*, *NOD2*, *NLRC3*, and *NLRC5*) clustered together, and the remaining two genes (*NLRX1* and *CIITA*) clustered separately. Compared with the traditional species tree, six genes of the *NLR* gene family could be used to distinguish species well above the family level. For example, within Mustelidae, Eurasian otters clustered with sea otters in the *NOD2*, *NLRC5*, and *CIITA* gene trees; Eurasian otters and American minks clustered together in the *NLRX1* gene tree; Eurasian otters and mink bears clustered together in the *NOD1* gene tree; and Eurasian otters clustered together with ferrets in the *NLRC3* gene tree.

The phylogenetic trees of *RLR* (Figure S2) and *CLR* (Figure S3) gene families were divided into three large branches, and the gene and species trees in each branch showed similar topological structures. Overall, the *PRR* genes were found to be relatively conserved during evolution and could be used as molecular markers to study the evolutionary status of a species.

### 3.3. Analysis of PPR Gene Selection Pressure

#### 3.3.1. Site Model Analysis

**Figure 5:**
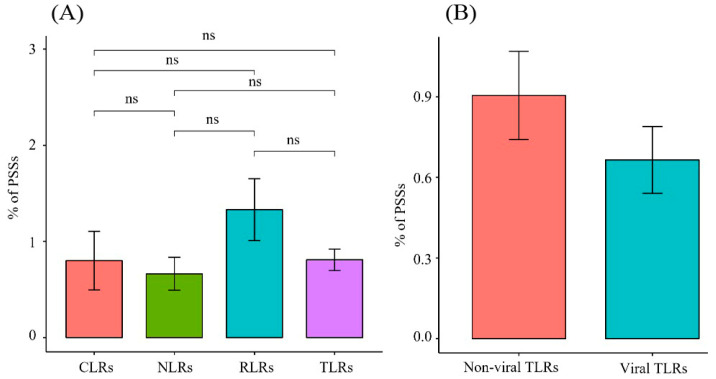
Proportion of positive-selection sites of different gene families in PRRs. (**A**) Comparison of the proportion of positive-selection sites between different gene families in PRRs. (**B**) Comparison of the proportion of positive-selection sites between non-viral and viral TLRs.

The M8 model outperformed the M7 model for all *PRR* genes, except for *TLR7* and *NOD1*. The data indicated that the *PRR* genes were positively selected during the evolution of Carnivora species. While analyzing the 22 PRR genes, four ML methods were used to screen 507 candidate positive-selection sites (Table S4), and the distributions of each gene were determined. In the *TLR* gene family, 236 candidate positive-selection sites were screened, and 71 were detected using more than two ML methods. *TLR4* had the most positive-selection sites (12). In addition, 44 positive-selection sites were identified in the non-viral *TLR* gene family, and 27 positive-selection sites were identified in the viral *TLR* gene family. The proportion of positive-selection sites in the family of non-viral *TLR* genes (0.9%) was higher than that of viral TLRs (0.67%); however, the *p*-value was not significant (Table 1, Figure 5). In the *RLR* gene family, the proportion of positive-selection sites was higher than that of other gene families (0.74–1.85%). The number of positive-selection sites (19) in *MDA5* was the largest among all the *PRR* genes (Table 1, Figure 5).

#### 3.3.2. Branch-Model Analysis

**Figure 6:**
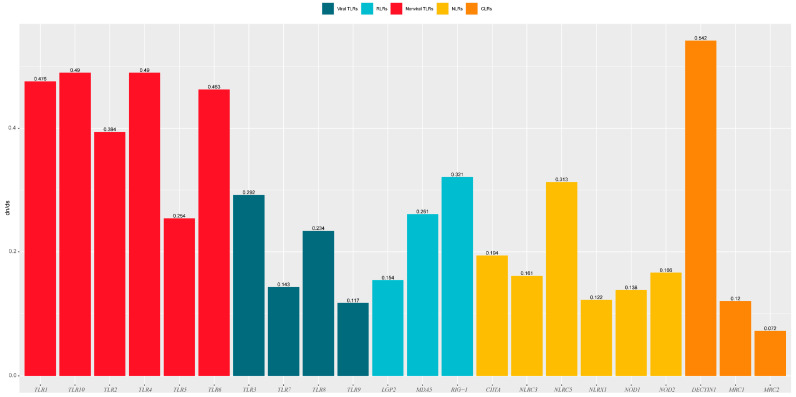
Synonymous/non-synonymous mutation ratio (dN/dS) of different genes in PRR genes.

**Figure 7:**
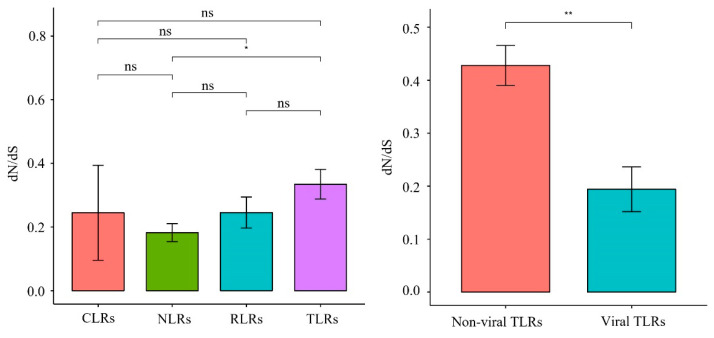
Synonymous/non-synonymous mutation ratio (dN/dS) of PRRs between different groups, * represents significant difference, ** represents extremely significant.

One-ratio (model A) analysis revealed that the ω values of the 22 PRR genes ranged from 0.072 to 0.542 (Figure 6, Table S5), indicating that these 22 genes were subjected to purifying selection during the evolution of carnivores. The comparison of the evolutionary rates of different gene families (Figure 7) revealed that the *TLR* gene family had a significantly higher (*p* = 0.015) evolutionary rate than the NLR gene family, which had the lowest evolution rate. Among the *TLR* gene family, non-viral *TLRs* showed a faster evolutionary rate than viral *TLRs* (*p* = 0.0044).

Free-ratio model (model B) analysis was a better model for some genes (*TLR4*, *TLR5*, *TLR8*, *TLR9*, *LGP2*, *NOD1*, *NLRC3*, *NLRC5*, *NLRX1*, *CIITA*, *MRC1*, and *MRC2*) than model A (Table S5). We filtered out results with a dN:dS ratio of >1; however, results with dS = 0 and the positively selected positions are marked on the species tree (Figure S4). The results showed that numerous positive-selection signals were distributed in the Ursidae, Canidae, and Felidae families and were more evident in brown bears, domestic dogs, wolves, and other species, suggesting that PRR genes undergo different evolutionary patterns in different Carnivora groups.

*TLR10* was detected as a pseudogene in seven species (*Lutra*, *Enhydra lutris*, *Pteronura brasiliensis*, *Neovison vison*, *Mustela putorius furo*, *Ursus americanus*, and *Cryptoprocta ferox*). To test whether the pseudogene status resulted from a relaxation of selection pressure, we deleted the stop codons in the *TLR10* genes of these seven species, reconstructed a dataset with *TLR10* of the other 36 Carnivora species, and performed branch-model analysis. Selection pressure analysis (Table 2) revealed that model A was significantly better than model B, indicating that *TLR10* underwent strong purifying selection in carnivores. Model C (two-ratio, assuming that the foreground branch containing the *TLR10* pseudogene and the background branch with normal *TLR10* have different ω values) outperformed model A.

Specifically, the ω value of the foreground branch (ω_2_ = 0.96503) was greater than that of the background branch (ω_1_ = 0.48586), suggesting that the seven species containing the pseudogene (*TLR10*) were subjected to different selection pressures than the other 36 Carnivora species. Significant relaxation of the selective pressure was observed in the branches of the seven species that contained pseudogenes. To further test whether the selective pressure was completely removed from the seven clades containing *TLR10* pseudogenes, we compared model D (two-ratio model with the ω value of the foreground clade set to 1) with model C. No significant difference was observed between the two models (*p* = 0.8129), indicating that the selection pressure of the seven pseudogenes was relaxed. Therefore, the *TLR10* pseudogenes in these seven species may represent a recent event.

#### 3.3.3. Branch-Site Model Analysis

The pathogenic environment faced by semi-aquatic species may have undergone greater changes than that of other terrestrial animals. To determine whether positive selection occurred in semi-aquatic species, we set all semi-aquatic Carnivora taxa as foreground clades and used the branch-site model for analysis. The branch-site model showed that *TLR2*, *TLR4*, *NLRC5*, and *DECTIN1* were positively selected in semi-aquatic Carnivora species (Table 3).

#### 3.3.4. Clade Model Analysis

The CmC model was used to detect the evolution rate of 22 PRR genes in two partitions of carnivore habitats, three partitions of feeding habits, and two partitions of flocking behaviors (Figure 1). Using the CmC model, significant differences were observed in all 22 PRRs between the different partitions in each category, except for the habitat partitions for TLR4; diet partitions of *MDA5* and *NLRC5*; habitat and diet partitions for *TLR5*; habitat and flocking partitions of TLR7; and all partitions of *TLR8*, *NOD1*, and *NOD2*. We compared the omega rates calculated in the CmC analyses considering both the type/family of PRR and the ecological niche (for instance, comparing the rates of viral and non-viral PRR for semi-aquatic species, comparing the rates for species with different dietary habits) in Figures S5 and S6. These results suggest that different PRRs also differ significantly in different habitat types, and TLR and RLR gene families have faster evolutionary rates in semi-aquatic groups, while NLR and CLR show opposite trends.

### 3.4. Annotation of the Positively Selected Sites of Three-Dimensional Protein Structures

**Figure 8:**
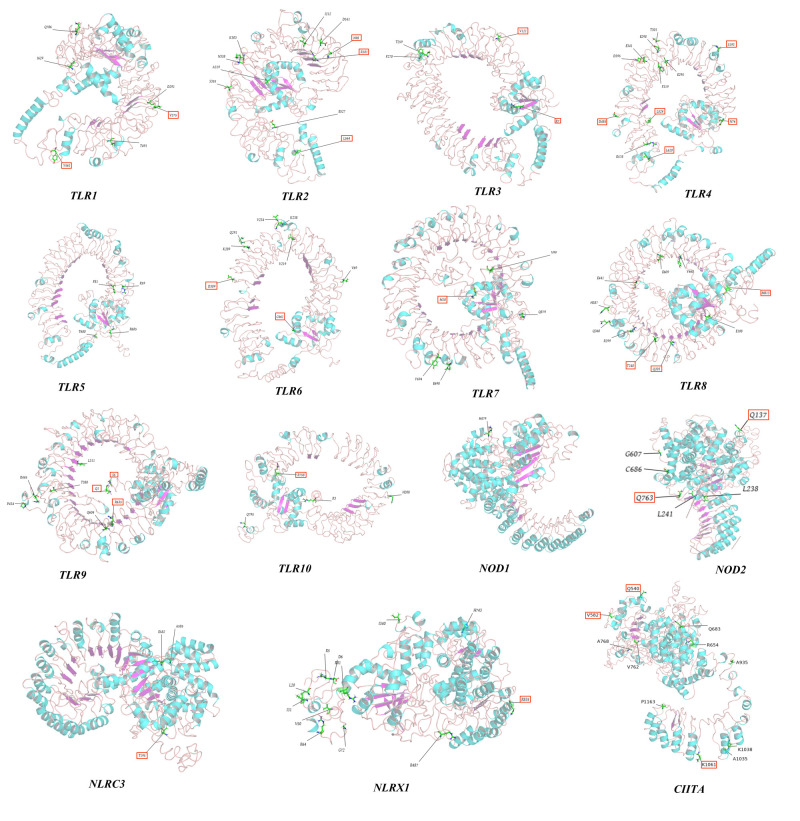
The 3D structures of TLRs and NLRs with positively selected sites marked.

In this study, PAML and Datamonkey software were used to analyze the sequences. The input sequences were deleted in regions with gaps. The positions of positive-selection sites were restored in the original sequence using the protein sequences and positions of Eurasian otter PRR genes as references (*TLR10* was based on the canine sequence). Most of the positive-selection sites in *MDA5 (12/19)*, *RIG-I (6/10)*, and *NLRC5 (9/14)* were located in their associated pathogen-binding and pathogen-recognition sites (Table S6). All positive-selection sites identified through screening were annotated on the three-dimensional protein structure of PRR genes to further evaluate their functional significance (Figure 8 and Figure S7).

## 4. Discussion

### 4.1. Relaxed Selective Pressure of the TLR10 Gene

Any PRR gene losses or pseudogenization may have important effects on the immune response of the body. In this study, it was found that there were some missing genes and pseudogenes in the Carnivora species (Figure 2). One of the reasons for this may be the poor quality of gene sequencing and assembly errors, and the real existence of these genes in species should be verified by PCR in later stages. *TLR10* was detected as a pseudogene in seven Carnivora species, and selection pressure was significantly relaxed in the lineages with the pseudogene (Table 2). We speculate that the relaxed selective pressure for *TLR10* genes in the seven carnivores may relate to redundant gene functions. *TLR10* is the only inhibitory receptor in the TLR gene family, and similar to *TLR1* and *TLR6*, it can both bind *TLR2* and mediate immune responses to inflammation caused by bacteria in the body [25]. Therefore, we speculate that a “functional compensation” mechanism may be associated with the *TLR* gene family. Although *TLR10* has become a pseudogene in seven Carnivora species, the possibility that other *TLR* genes (*TLR1* and *TLR6*) serve as function substitutes still remains. Previous data showed that redundancy, gene loss, or altered ligand specificities of certain immune receptor functions may result from changes in environmental pathogens [26]. Among the seven species with *TLR10* pseudogenes, three species of the otter subfamily and American mink adapted to semi-aquatic environments, where they may have faced more complex pathogenic environments than terrestrial species. Therefore, *TLR10* pseudogenization might also be related to the living environments of the associated species.

### 4.2. Differences in the Evolution of Different Carnivora PRR Subfamilies

Previous findings have revealed that, overall, *PRR* genes are evolutionarily subject to purifying selection pressure owing to different selection pressures [27,28]. Focusing on Carnivora species, we found that the ω values of 22 *PRR* genes were all significantly less than 1 (0.072–0.542) (Figure 6), indicating that they were also affected by purifying selection during Carnivora evolution. The sequences of pathogenic microorganisms are relatively conserved, and mutations that cause structural changes are mostly deleterious. In this study, we observed that conserved *PRR* genes were subjected to purifying selection pressure, which may have facilitated the removal of harmful mutations and helped maintain the effective identification of specific pathogens. Our analysis of the evolutionary rates of different gene families (Figure 7) revealed that the *TLR* gene family underwent relatively rapid evolution, where the mutation rate of the *TLR* gene family was significantly higher than that of the *NLR* gene family with the lowest evolutionary rate (*p* = 0.015), and no significant differences were observed when compared with other gene families within the *NLR* gene family. Previous findings showed that the *NLR* gene family has a lower evolutionary rate than other PRRs [28], indicating that NLRs have been highly conserved during evolution and show higher tendencies to clear deleterious mutations. *NLR* proteins are generally expressed in the cytoplasm, although some *TLRs* are located on the plasma membrane. The results of this study suggest that *NLRs* are more conserved during evolution than TLRs, which may be related to the relatively narrow recognition of PAMPs by NLRs and the relative inability to tolerate multiple mutations. In addition, in the *TLR* gene family (Figure 7), non-viral *TLRs* showed a faster evolutionary rate than viral *TLRs* (*p* = 0.0044). Non-viral TLRs primarily recognize bacteria in the plasma membrane, whereas viral TLRs primarily recognize viruses in cells. Wlasiuk et al. also found that viral-type TLRs are more conserved than bacterial-type *TLRs* in primate studies, suggesting that the plasma membrane may be more vulnerable to pathogens than cells [29], consistent with our current results.

### 4.3. Carnivora PRR Genes Universally Underwent Positive Selection

Although PRR genes comprise a class of functionally constrained genes, the evolutionary process was interspersed with positive selection. In this study, 22 PRR genes of different Carnivora species were analyzed using four ML methods using PAML and Datamonkey software. All PRR genes were found to have positively selected loci that could be detected using at least two methods (Figure 2). Pathogenic microorganisms continuously exert different selection pressures on the host immune system through continuous reproduction, mutation, and evolution [24]. In this study, numerous positive-selection sites were detected in PRR genes, indicating that during the “arms race” between the host and exogenous pathogenic microorganisms, co-evolution may have occurred with pathogenic microorganisms to better resist the threat of pathogenic microorganisms. Adaptive evolution in any species may exert selective pressure on the other species, and this interrelationship drives co-evolution between both species [30].

In the *TLR* gene family, the proportion of positive-selection sites in the non-viral TLR (0.9%) gene family was higher than that in the viral *TLR* (0.67%) gene family (Figure 5), which also indicates that bacterial *TLRs* have a faster evolution rate than viral TLRs [24,29,31]. Among the non-viral *TLRs*, *TLR4* and *TLR2* had many positive-selection sites (Table S4). Both the *TLR2* and *TLR4* proteins are located on the cell membrane, and *TLR2* can identify many bacteria, fungi, and endogenous substances [32]. In terms of function, *TLR4* and *TLR2* not only recognize LPSs of familiar Gram-negative bacteria but also monitor fungi, viruses, and protozoa [33]. From the perspective of signaling pathways, *TLR4* and *TLR2* are the most complex, can rely on the *MYD88* signaling pathway to transform into the TRIF pathway, and need to interact with co-receptor *MD2* to function [34,35]. Therefore, *TLR4* has the most positive-selection sites to cope with such diversified functional characteristics. Studies have revealed that abnormalities in *TLR4* and *TLR2* are associated with cardiovascular and cerebrovascular diseases, obesity, diabetes, tumors, and metabolic diseases [36]. Previous studies have also revealed many positive-selection sites in the *TLR2* and *TLR4* genes of birds, mammals, and reptiles [29,31,37,38,39]. The results of this study also support the fact that *TLR2* and *TLR4* may have important roles in recognizing more exogenous pathogenic bacteria during evolution than other *TLRs* and have more positive-selection sites, which may be related to different ligands and ways of recognizing ligands.

Among viral *TLRs*, *TLR8* showed the most positive-selection sites, consistent with previous studies on bat and mammalian viral TLRs [39,40]. The *TLR8* gene is primarily expressed in the lungs and peripheral blood erythrocytes and is located next to *TLR7* on the X chromosome. *TLR8* can identify single-stranded RNA (ssRNA) and ssRNA viruses, such as the influenza virus, Sendai virus, and Coxsackie B virus [41,42]. The extensive positive selection of *TLR8* in different species or taxa suggests that it may have played an important host-defense role against viral threats. Among the RLR gene family, strong positive-selection signals were detected for both *MDA5* and *RIG-I,* consistent with previous findings in mammals and reptiles [28,43]. *MDA5* and *RIG-1* share highly homologous structures and similar signaling pathways but elicit different responses to viral antigens. *RIG-I* primarily identifies relatively short double-stranded RNAs (dsRNAs; up to 1 Kb) in the Paramyxovirus and Rhabdoviridae families, and *MDA5* primarily detects long dsRNAs (>2 Kb) from picornaviruses, such as encephalomyocarditis virus [5,44]. During evolution, long dsRNAs potentially exerted greater selection pressure on Carnivora, thereby promoting co-evolution with *MDA5* (which identifies long dsRNAs and pathogens). In addition, the results of this study showed that most positive-selection sites present in *MDA5 (12/19)* and *RIG-I (6/10)* were located in functional protein domains related to pathogen binding and recognition (Tables S1 and S3), which may have conferred stronger antiviral abilities to both genes. Among the *NLR* gene family, *NOD1* primarily identifies meso-diaminopimelic acid expressed in Gram-negative bacteria, whereas *NOD2* primarily identifies the intracellular muramyl dipeptide [45,46]. However, in this study, fewer positive-selection sites were detected in *NOD1* and *NOD2*, whereas strong positive-selection signals were detected in NLRC5 (Table 2), most of which (9/14) are located in or near the functional protein domains (Table S6). Previous data showed that *NLRC5* has the highest molecular weight of the *NLR* family and is widely expressed in various organs and tissues. *NLRC5* can generate innate immunity against viruses by modulating interferon activity and regulating the expression of major histocompatibility class I [47,48,49]. We speculate that during evolution, owing to its extensive expression and multiple biological functions, *NLRC5* faced stronger pathogenic pressure than other *NLRs*, resulting in greater positive selection of its functional protein domains. In this study, we found that among members of the *CLR* gene family, the *DECTIN1* gene had a relatively high proportion of positive-selection sites (1.22%); however, only three positive-selection sites were detected by two ML methods, which may be related to the relatively short gene sequence (246 amino acids).

### 4.4. Evolutionary Relationship between Niches and PRR Genes

During carnivore evolution, different lineages had different feeding habits, flocking behaviors, and habitat types. The genetic evolution of immunity-related gene families caused by differences in habitats, feeding habits, and flocking behaviors has gradually attracted widespread attention [50]. Previous findings show that different dietary habits lead to differences in the gut microbiota, which in turn regulate the expression of immunity-related genes [51]. Therefore, an important question is about the relationship that exists between diet and immunity-related genes. Our CmC results (related to different clades) showed that, except for the *TLR5/8*, *NDA5*, *NOD1/2*, and *NLRC5* gene models (which were not significant), the remaining PRR genes underwent different evolutionary rates in association with each of the three feeding habits (Table S7). The living environment of the host determines the pathogenic species to which it is exposed [50,52]. Except for the *TLR4/5/7/8* and *NOD1/2* genes, the remaining PRR gene models showed significant differences between semi-aquatic and terrestrial taxa (Table S7), suggesting that different PRRs underwent markedly different evolution in different types of habitats. Animals that live in groups may be more susceptible to parasitic infections than solitary animals, thereby acquiring higher risks for certain diseases [53]. In this study, aside from *TLR7/8* and *NOD1/2*, the other PRR genes showed different evolutionary rates with different clustering behaviors (Table S7), suggesting that the clustering behavior also affects PRR evolution. In conclusion, all PRRs except *TLR8* and *NOD1/2* genes were divided into at least one species group under three different ecological niches, indicating that different ecological niches may have jointly driven the evolution of genes in carnivore PRRs

The results of the branch-point model showed that *TLR2*, *TLR4*, *NLRC5*, and *DECTIN1* had detectable positive-selection sites among the semi-aquatic taxa, and all models showed significant differences (Table 3). Bacteria and viruses in aquatic environments (freshwater and marine) may significantly differ from the closest terrestrial relatives [52]; therefore, amphibians may face greater selection pressure from pathogens than purely terrestrial animals. Shishido et al. found that adaptive amino acid changes occurred in the heterodimeric domain formed by the cetacean *TLR4* gene and its ligand, *MD-2* [54]. Furthermore, Shen et al. found that the *TLR4* gene showed a strong positive-selection signal in early cetacean ancestors returning from the land to the ocean and a rapid radiation stage for Delphinidae [55]. Shang et al. found that the *TLR2* gene has undergone different evolutionary rates in semi-aquatic and terrestrial groups of reptiles, with adaptive evolutionary sites in the semi-aquatic group [43]. Chen et al. found a strong positive-selection signal in the *MDA5* genes of reptiles, and adaptive amino acid changes occurred in semi-aquatic taxa [28]. Therefore, habitats appear to play important roles in driving changes in immunity-related, gene-specific amino acid sites. The results of this study suggest that the aforementioned semi-aquatic, group-specific amino acid changes may have promoted the effects of *TLR2*, *TLR4*, *NLRC5*, and *DECTIN1* against pathogens in aquatic environments, thereby facilitating the adaptation to aquatic environments. In addition, we also detected the presence of positive-selection sites for *TLR1*, *TLR4*, *TLR6*, *TLR9*, *LGP2*, and *DECTIN1* genes in terrestrial taxa (*p* < 0.05) (Table S8), suggesting that alterations in the amino acid sites of these genes may also promote better adaptation of terrestrial taxa to pathogens in terrestrial environments.

## 5. Conclusions

In summary, we systematically identified 22 PRR genes (out of a total of 946 gene sequences) and performed phylogenetic and evolutionary analyses in Carnivora species. The phylogenetic analysis results showed that PRR genes were relatively conserved during the evolutionary process and could be used as molecular markers to study the evolutionary status of species. Evolutionary analysis revealed that PRRs were under purifying selection pressure in the whole carnivore species, and the overall evolutionary rate of non-viral TLRs was significantly higher than that of viral TLRs. In addition, different genes and gene families show different evolution rates, which may be related to the different functions they perform. Lastly, we found that diets, clustering behavior, and habitat type may drive the evolution of PRR genes in Carnivora species. The positive selection loci detected in the *TLR2*, *TLR4*, *NLRC5*, and *DECTIN1* genes of the semi-aquatic group of Carnivora may be related to the adaptation of this group to the semi-aquatic environment.

## Figures and Tables

**Table 1:** Positively selected sites of PRRs detected by different ML methods in Carnivora.

Gene	Two ML	Three ML	Four ML	% of Sites (≥2 ML)
*TLR1*	5	1	0	0.76
*TLR2*	6	3	1	1.26
*TLR3*	2	1	1	0.44
*TLR4*	5	4	3	1.44
*TLR5*	4	0	0	0.47
*TLR6*	8	0	0	1.01
*TLR7*	5	0	0	0.48
*TLR8*	8	2	0	0.96
*TLR9*	4	1	3	0.78
*TLR10*	2	0	2	0.49
*LGP2*	1	1	3	0.74
*MDA5*	15	2	2	1.85
*RIG-I*	10	0	3	1.4
*DECTIN1*	3	0	0	1.22
*MEC1*	1	2	0	0.21
*MRC2*	10	1	3	0.97
*NOD1*	1	0	0	0.1
*NOD2*	6	0	0	0.59
*NLRX1*	12	0	0	1.23
*CIITA*	6	5	0	0.91
*NLRC3*	3	0	0	0.29
*NLRC5*	6	6	4	0.86

**Table 2:** Selection pressure test of *TLR10* in Carnivora species through branch model.

Model	np	*LnL*	ω for Branch	Model Compared	2△LnL	*p*-Values
A: All the branch has same ω	86	−10,469.187	ω = 0.54602			
B: All the branch has same ω = 1	85	−10,516.988	ω = 1	B vs. A	95.6024	<0.01
C: The branches with a pseudogenized *TLR10* have the ω_2_; other branches have a ω_1_	87	−10,459.788	ω_1_ = 0.48586 ω_2_ = 0.96503	C vs. A	18.7986	<0.01
D: The branches with a pseudogenized *TLR10* have the ω_2_ = 1; other branches have a ω_1_	86	−10,459.816	ω_1_ = 0.48608 ω_2_ = 1	D vs. C	0.056	0.8129

**Table 3:** Positive selection using branch-site model of PRR genes in the Carnivora species.

Gene	Models	np	LnL	LRT *p*-Values	Positive
*TLR1*	Model 1 null	74	−8759.05	0.237	NA
	Model 1	75	−8758.35		
*TLR2*	Model 1 null	86	−10,345.5	0.000801	301**V 454R
	Model 1	87	−10,339.9		
*TLR3*	Model 1 null	88	−11,802.1	0.371	NA
	Model 1	89	−11,801.7		
*TLR4*	Model 1 null	86	−11,602.5	0.0000481	319F 395D 604*V
	Model 1	87	−11,594.3		
*TLR5*	Model 1 null	80	−16,674.5	1	Null
	Model 1	81	−16,674.5		
*TLR6*	Model 1 null	80	−11,509.7	0.0727	NA
	Model 1	81	−11,508.1		
*TLR7*	Model 1 null	82	−11,828.6	1	NA
	Model 1	83	−11,828.6		
*TLR8*	Model A null	80	−14,115.5	1	NA
	Model 1	81	−14,115.5		
*TLR9*	Model 1 null	78	−15,197.2	0.842	Null
	Model 1	79	−15,197.2		
*TLR10*	Model 1 null	74	−9133.87	1	NA
	Model 1	75	−9133.87		
*LGP2*	Model 1 null	86	−9825.26	1	Null
	Model 1	87	−9825.26		
*MDA5*	Model 1 null	82	−12,345.3	0.182	NA
	Model 1	83	−12,344.4		
*RIG-I*	Model 1 null	86	−11,151.2	0.000962	NA
	Model 1	87	−11,145.7		
*NOD1*	Model 1 null	84	−11,348.6	0.431	Null
	Model 1	85	−11,348.3		
*NOD2*	Model 1 null	80	−7349.27	0.48	NA
	Model 1	81	−7349.02		
*NLRC3*	Model 1 null	72	−13,289.7	0.842	NA
	Model 1	73	−13,289.7		
*NLRC5*	Model 1 null	66	−25,540.2	0.0021	931Q 1365*E 1366E
	Model 1	67	−25,535.5		
*NLRX1*	Model 1 null	84	−13,104.4	1	NA
	Model 1	85	−13,104.4		
*CIITA*	Model 1 null	78	−11,736.1	1	NA
	Model 1	79	−11,736.1		
*MRC1*	Model 1 null	70	−16,562.3	0.527	NA
	Model 1	71	−16,562.1		
*MRC2*	Model 1 null	72	−14,544.4	0.439	Null
	Model 1	73	−14,544.1		
*Dectin1*	Model 1 null	80	−3766.48	0.00414	115*F
	Model 1	81	−3762.37		

“NA” means no positive-selection site; “Null” means the model is not significant; all genes use Eurasian otter as the reference sequence; * represents significant difference, ** represents extremely significant.

## Data Availability

Not applicable.

## Supplementary Materials

The following supporting information can be downloaded at: https://www.mdpi.com/article/10.3390/ani12233331/s1, Figure S1: Accession number of PRR genes used in this study; Figure S2: Phylogenetic tree of RLR genes in the Carnivora species; Figure S3: Phylogenetic tree of CLR genes in the Carnivora species; Figure S4: Evidence of positive selection estimated by free ratio in different PRR genes; Figure S5: Synonymous/non-synonymous mutation ratio (dN/dS) of PRRs between different groups in Clade model analysis. Figure S6: Synonymous/non-synonymous mutation ratio (dN/dS) of PRRs between viral and non-viral groups in Clade model analysis. Figure S7: The 3D structures of RLRs and CLRs with positively selected sites marked; Table S1: Genomes annotations information of 43 carnivora species; Table S2: The accession numbers of the sequences used as seed in the identification of PRR genes; Table S3: Gene information of 43 Carnivora species; Table S4: Positively selected sites of PRRs genes in Carnivora species; Table S5: Selection pressure test of PRRs in carnivora species through Branch model; Table S6: Positively selected sites predicted to affect function in PRRs genes based on their location; Table S7: Model comparison between CmC and NULL model for TRC PRRs genes; Table S8: Positive selection using branch-site model of PRRs genes in the Carnivora species.
