# Supplementary figures and images for: Evolutionary Impacts of Pattern Recognition Receptor Genes on Carnivora Complex Habitat Stress Adaptation
Source: Animals (Basel). 2022 Nov 28;12(23):3331. doi: 10.3390/ani12233331 (PMC9739989; doi:10.3390/ani12233331)

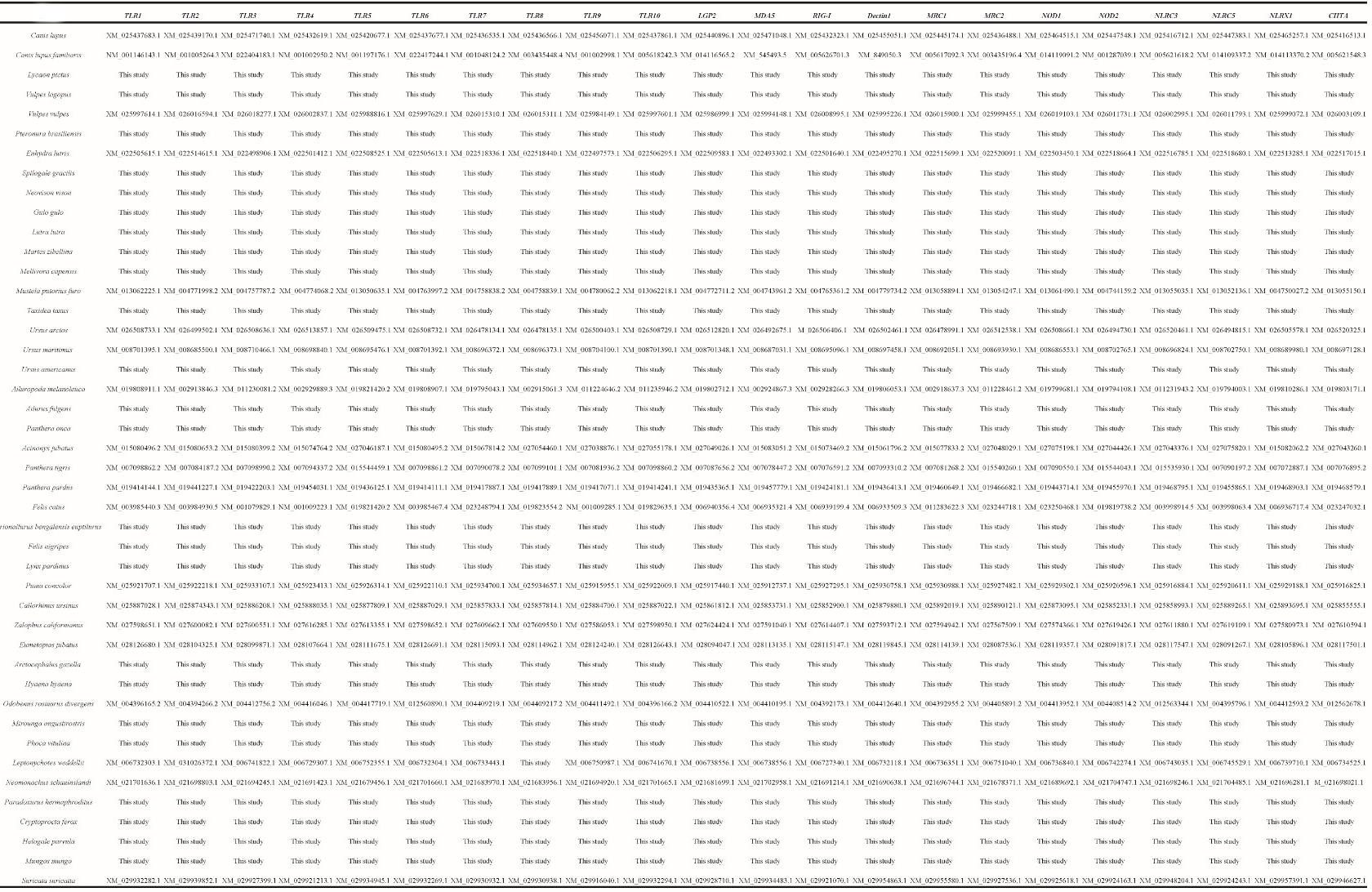

Supplement: Supplementary file 1 [file animals-12-03331-s001.zip › Supplementary Files/Figure S1.jpg]

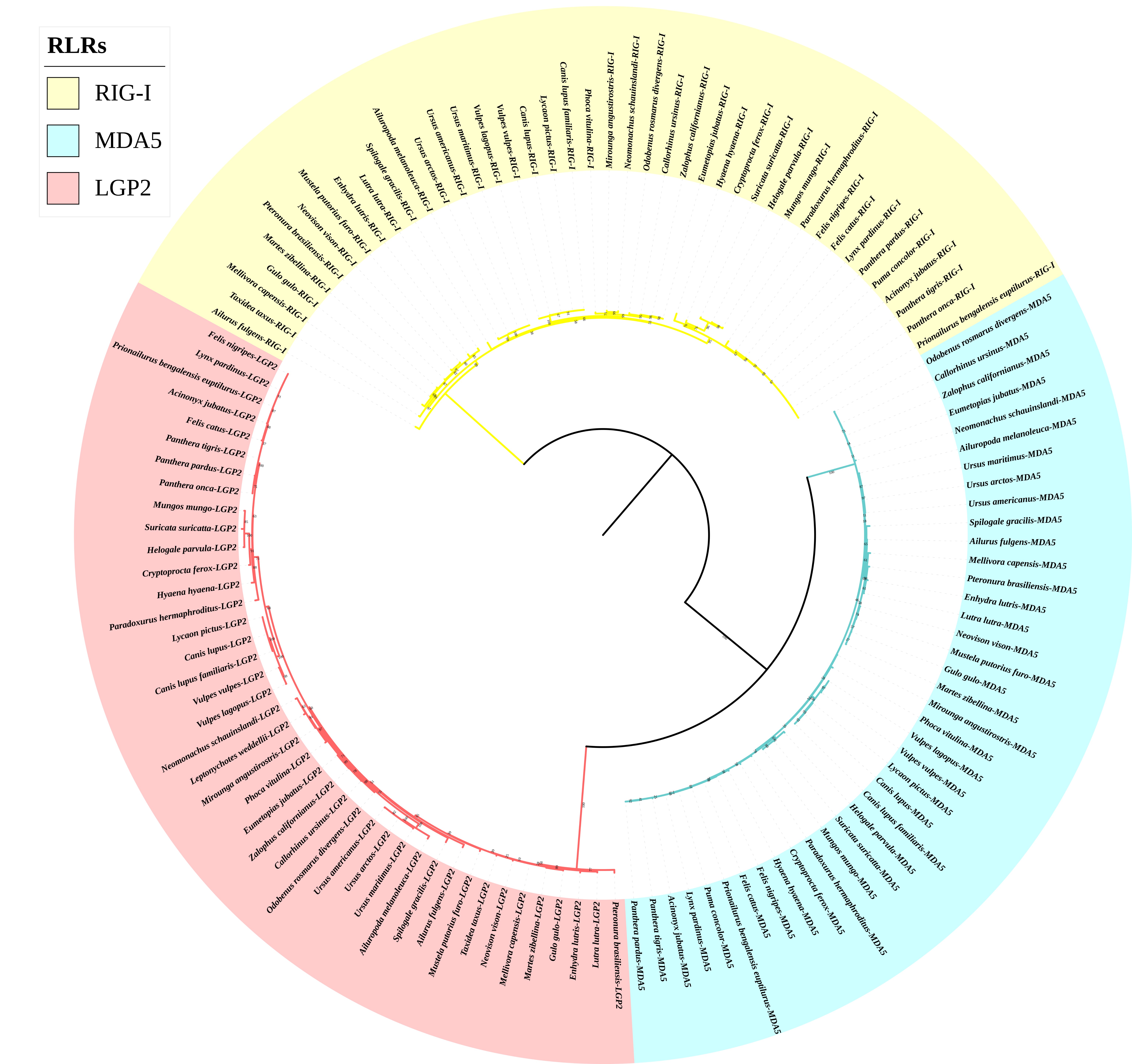

Supplement: Supplementary file 1 [file animals-12-03331-s001.zip › Supplementary Files/Figure S2.jpg]

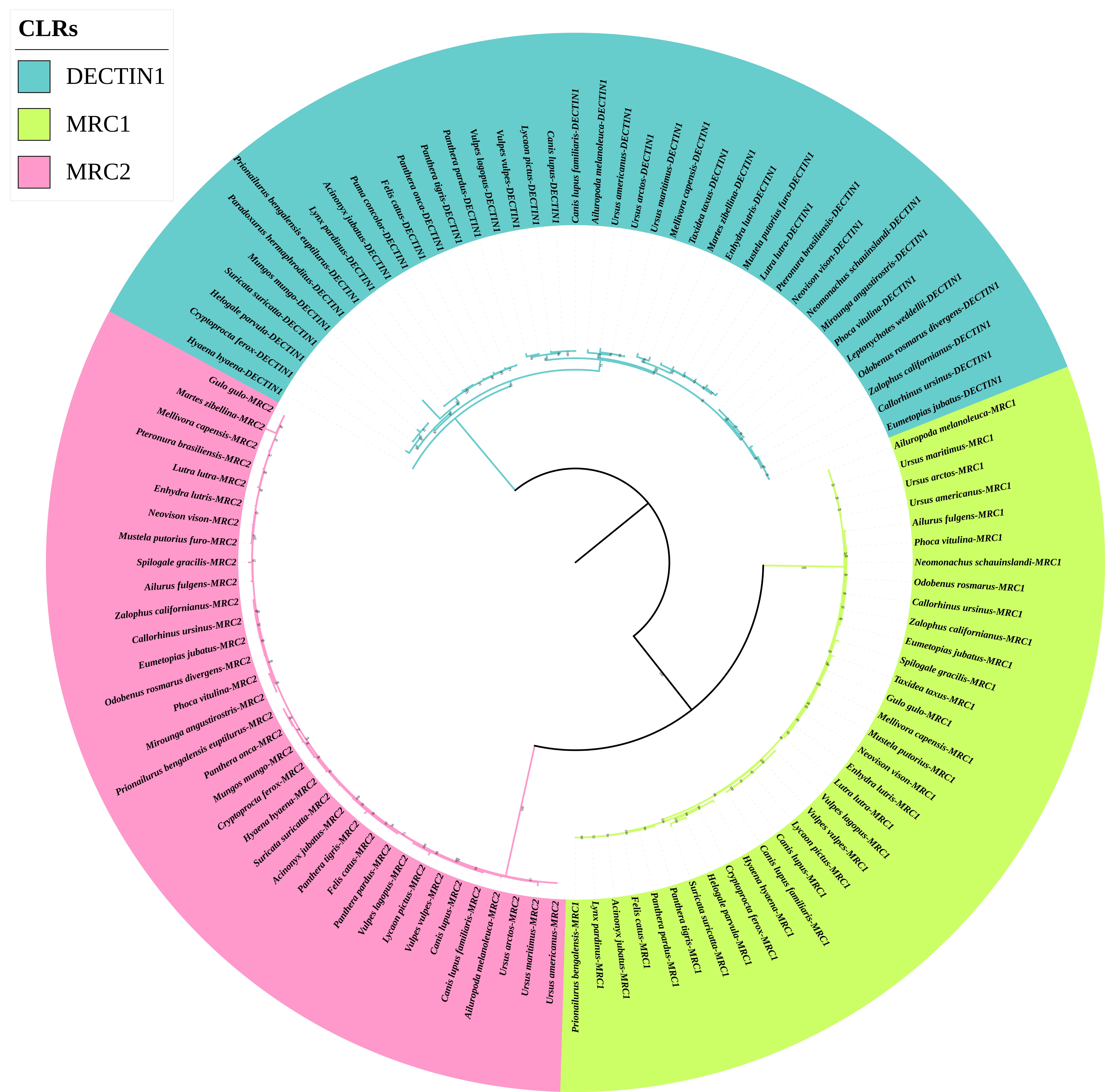

Supplement: Supplementary file 1 [file animals-12-03331-s001.zip › Supplementary Files/Figure S3.jpg]

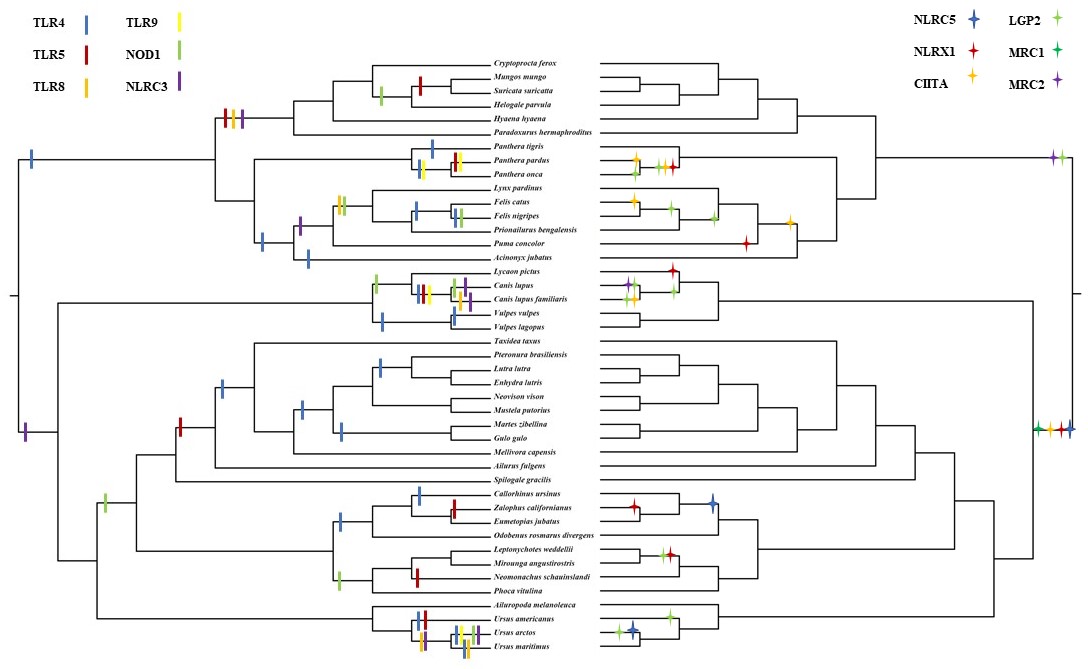

Supplement: Supplementary file 1 [file animals-12-03331-s001.zip › Supplementary Files/Figure S4.jpg]

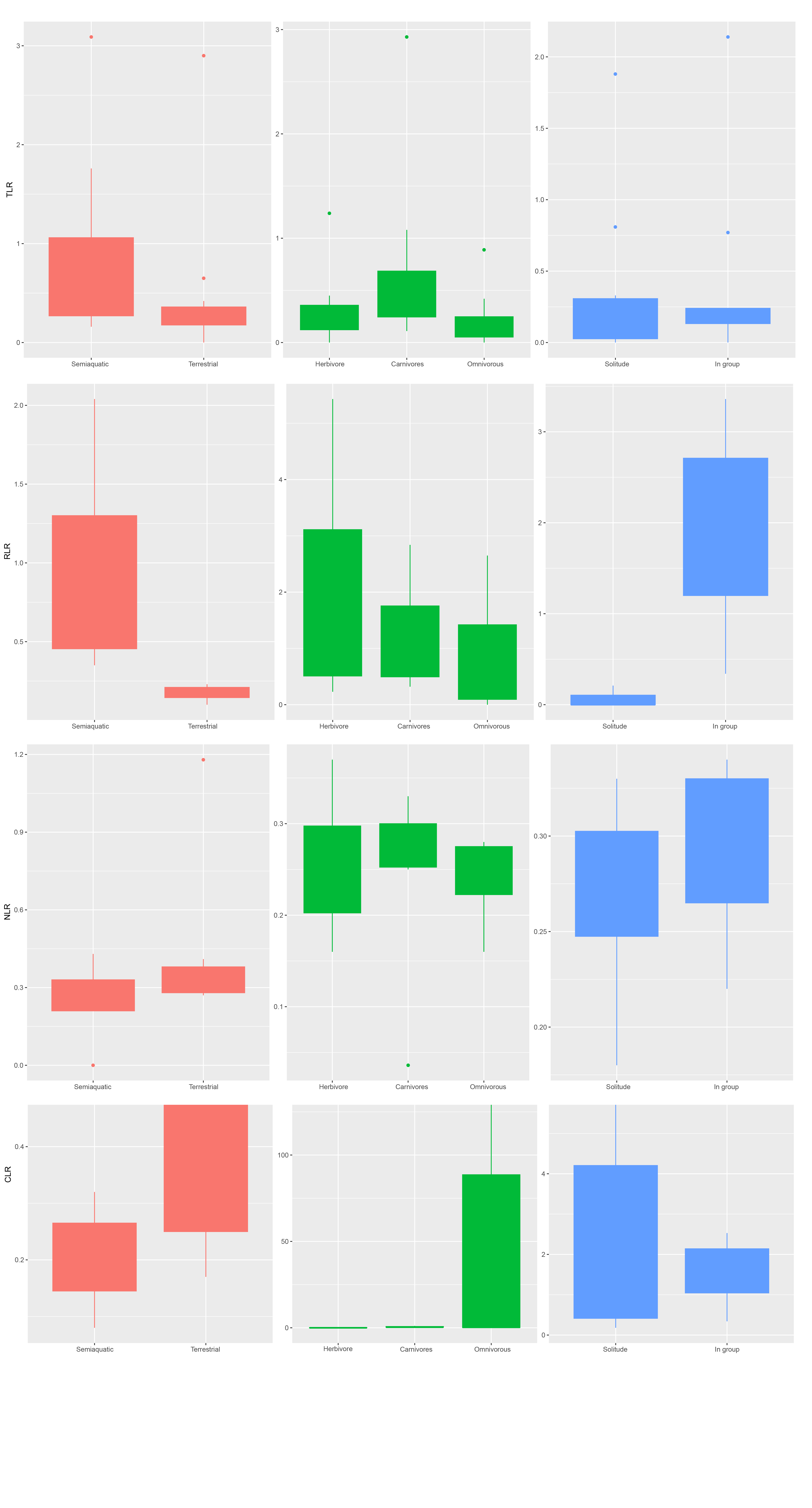

Supplement: Supplementary file 1 [file animals-12-03331-s001.zip › Supplementary Files/Figure S5.jpg]

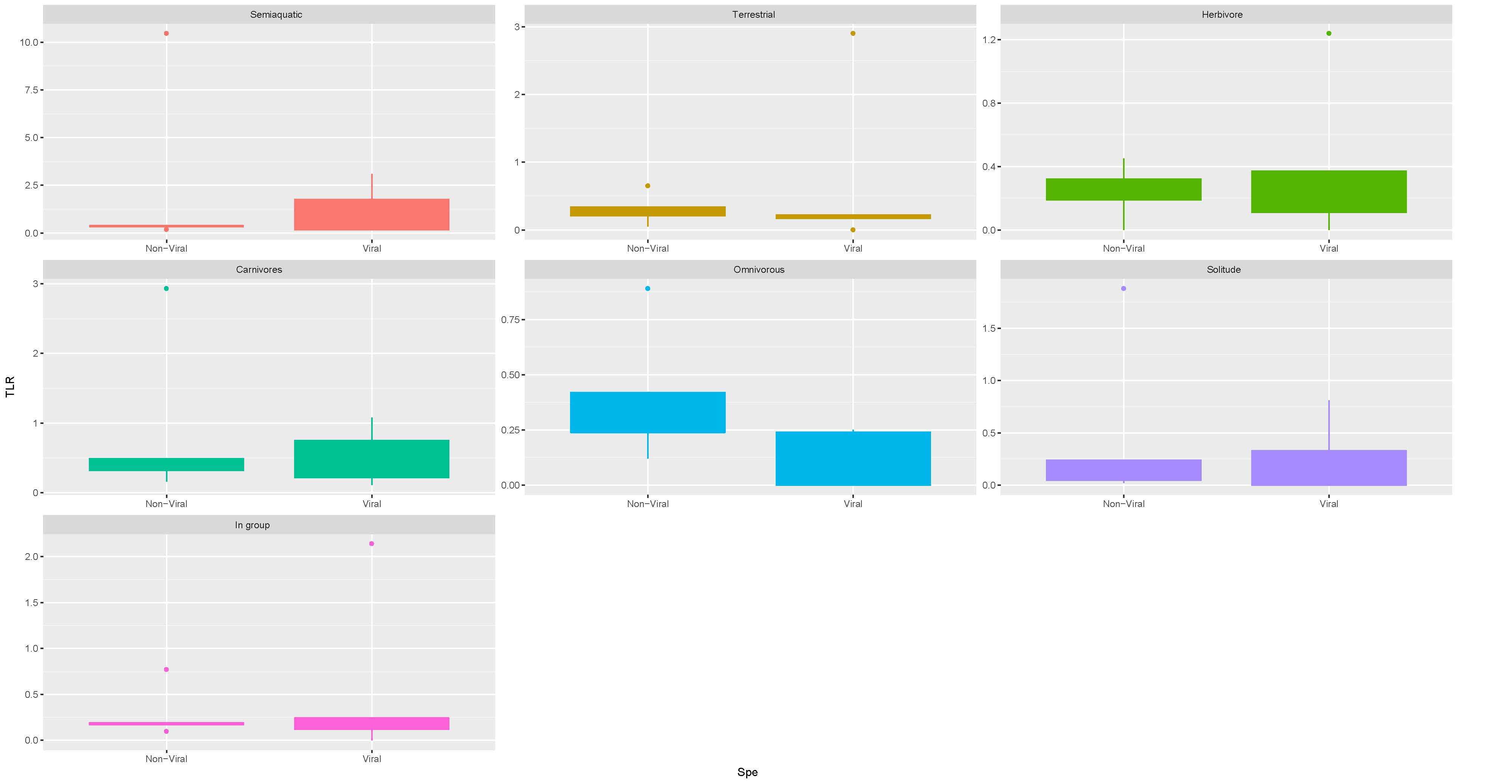

Supplement: Supplementary file 1 [file animals-12-03331-s001.zip › Supplementary Files/Figure S6.jpg]

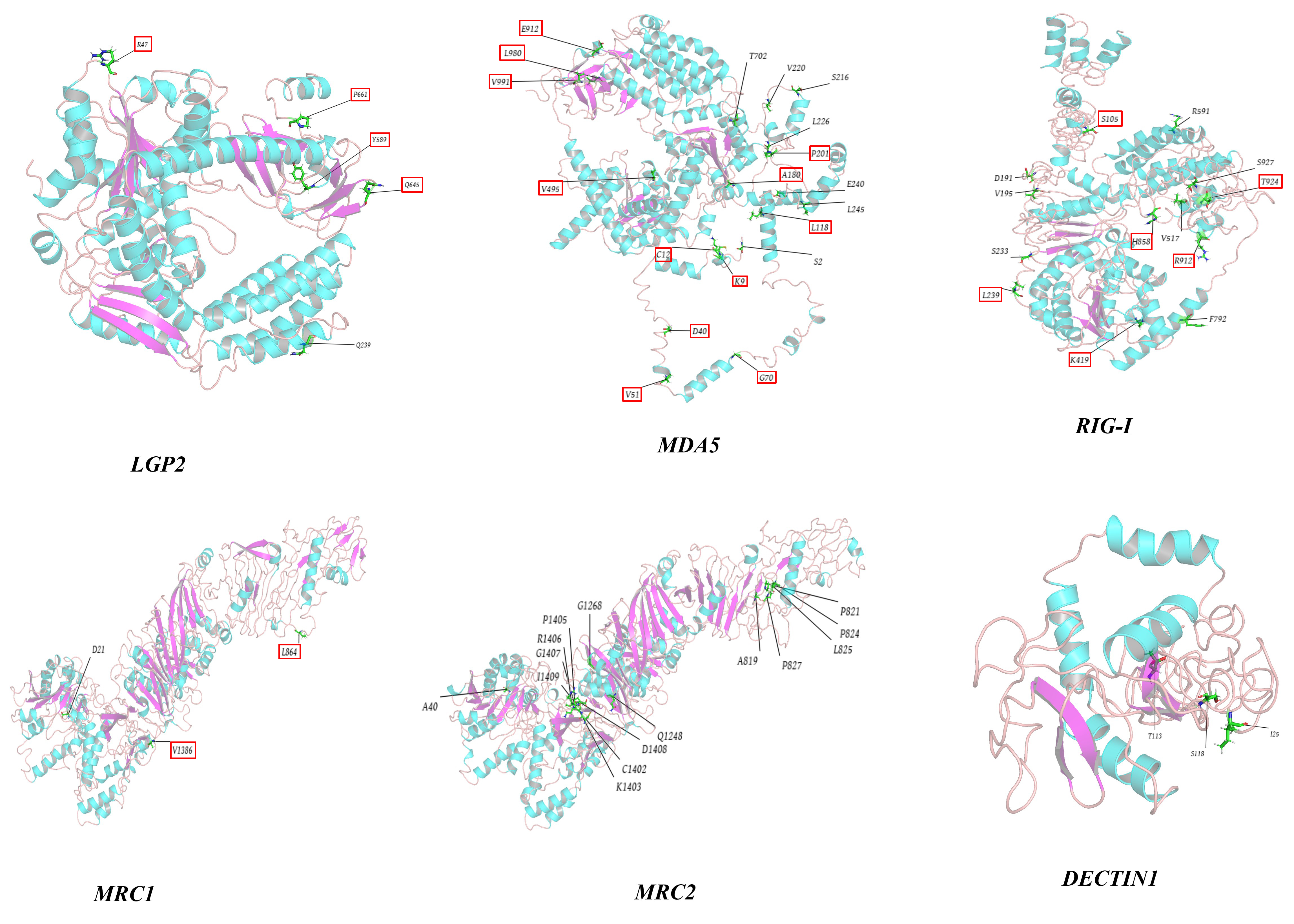

Supplement: Supplementary file 1 [file animals-12-03331-s001.zip › Supplementary Files/Figure S7.jpg]
